# Reprogramming Immune Cells for Enhanced Cancer Immunotherapy: Targets and Strategies

**DOI:** 10.3389/fimmu.2021.609762

**Published:** 2021-04-21

**Authors:** Yan Dong, Zhuo Wan, Xiaotong Gao, Guodong Yang, Li Liu

**Affiliations:** ^1^Department of Hematology, Tangdu Hospital, Fourth Military Medical University, Xi'an, China; ^2^Department of Biochemistry and Molecular Biology, Fourth Military Medical University, Xi'an, China

**Keywords:** cancer immune therapy, cell engineering, targets, strategies, immune cells

## Abstract

Cancer is one of the leading causes of death and a major public health problem all over the world. Immunotherapy is becoming a revolutionary clinical management for various cancer types. Restoration of aberrant immune surveillance on cancers has achieved markable progress in the past years by either *in vivo* or *ex vivo* engineering of the immune cells. Here, we summarized the central roles of immune cells in tumor progression and regression, and the existing and emerging strategies for different immune cell-based immunotherapies. In addition, the current challenges and the potential solutions in translating the immunotherapies into the clinic are also discussed.

## Introduction

Cancer incidence and mortality have been increasing since 2010, making cancer the leading cause of death and a major public health problem all over the world (1, 2). The traditional cancer therapies, such as surgery, radiotherapy, and chemotherapy, have difficulty in completely eradicating cancer cells. The emerging immunotherapy is revolutionizing the clinical management of multiple tumors (3).

Tumor microenvironment (TME) is infiltrated by immune cells, which together with stromal cells, contribute to tumor escape from host immune surveillance and its progression (4, 5). Generally, the immune cells in TME can be divided into two types: tumor-antagonizing and tumor-promoting immune cells. The tumor-antagonizing immune cells consist of CD8^+^ cytotoxic T cells, effector CD4^+^ T cells, natural killer (NK) cells, dendritic cells (DCs), M1-polarized macrophages, and N1-polarized neutrophils. In contrast, the tumor-promoting immune cells mainly consist of regulatory T cells (Tregs) and myeloid-derived suppressor cells (MDSCs). MDSCs could be further divided into two subtypes: the polymorphonuclear MDSCs (PMN-MDSCs) and the monocytic MDSCs (M-MDSCs). The PMN-MDSCs are morphologically similar to N2-polarized neutrophils, whereas M-MDSCs are similar to M2-polarized macrophages. Notably, the role of B cells in TME is relatively unclear and controversial, with both tumor-antagonizing and protumorigenic roles being reported (6).

Aberrant innate and adaptive immune responses are closely related to immunosuppression and tumorigenesis (7). During the early stages of tumor progression, natural killer (NK) cells and CD8^+^ T cells act as cytotoxic immune cells to recognize and kill tumor cells (8). However, there remains a subset of less immunogenic tumor cells that survived as the dominant cells afterward, which escaped immune surveillance (9, 10). As the tumor continuously grows, different kinds of immune cells adopt various ways to form the immune-suppressive microenvironment, which eventually weakens the tumoricidal effects (11).

The main mechanism of immunotherapy is to change the tumor microenvironment or the immune cells so that the immune system can achieve the purpose of killing tumors (5). Immunotherapies targeting accessory immune cells are considered as promising strategies against a variety of cancers (5, 11, 12). Here, we summarize the key roles of different immune cells in the tumor microenvironment and emerging immunotherapy strategies based on modulation of different immune cells. Future challenges and the possible solutions that translate the immunotherapies into clinical reality are also discussed.

## Chimeric Antigen Receptor T-Cell-Based Immunotherapy

Malignant progression stimulates adaptive immune responses and creates a subset of specific T cells that can precisely eliminate tumor cells (13, 14). However, with the development of tumors, tumor cells become less immunogenic and lose specific tumor antigens that activate adaptive response at the beginning of malignant progression (15, 16). Besides, low expression of class I MHC molecules on tumor cells also leads to the downregulation of CD8^+^ cytotoxic T lymphocytes (CTLs) and thus immunosuppression (17). Immune checkpoints, CTLA-4 and PD-1/PD-L1 are also blamed to suppress T-cell activity when bound by the corresponding ligands on tumor cells (18–20).

Chimeric antigen receptor (CAR) T cell technology is an innovative therapy, which harness the inherent capacity of the immune system to fight cancer selectively in an MHC-independent way. CARs are synthetic antigen receptors that include both antigen recognition moieties and T-cell activation signaling domains (21). A CAR consists of three major domains: ectodomain, transmembrane domain, and endodomain (22, 23). The ectodomain is exposed to the extracellular space with signal peptide, antigen recognition region, and spacer. The antigen recognition region is usually a single chain variable fragment (scFv) formed by fusing the variable portions of heavy and light chains of a monoclonal antibody with a flexible linker (24, 25). The scFv presents the function of identifying and binding tumor antigens with high affinity. A spacer functions as a connection between the antigen-binding domain and the transmembrane domain (26). The transmembrane domain is derived from most of the membrane-proximal components of the endodomain and consists of a hydrophobic alpha helix spanning the membrane, which is related to the stability of the receptor. CD3ζ serves as the most common component of the endodomain, which activates T cells after CAR binds the target antigen. Additionally, the CAR internal domain undergoes intergenerational changes, including one or more costimulatory domains, such as the commonly used CD28 and 41BB, to enhance the persistence and cytotoxicity of CAR-expressing cells (27, 28).

Based on the structure of the endodomain, CAR-T cells can be roughly divided into four generations (29). The first generation CARs mimicked the signals from endogenous T-cell receptor (TCR), which contains only one activating domain (usually a portion of the ζ chain in the TCR complex). Additional activating domains are then added in the next two generations, with more CAR-T-cell proliferation, stronger killing ability, and higher cytokine production. The fourth-generation CARs were typically characterized by the addition of IL-12 to the second generation of CAR-T (30). Currently, CAR-T have been tried to various types of cancer, including leukemia and solid tumors. Moreover, CAR-T could be even engineered to eradicate the cancer stem cells.

### CAR-T for Leukemia

CAR-T immunotherapy has produced a particularly successful clinical response in the treatment of hematologic malignancies. Up to now, three CAR-T cell products have been approved by the U.S. Food and Drug Administration, which all target CD19 antigen. CD19 is a cell-surface component of the B-cell receptor complex involved in B-cell activation, which expressed at high and stable levels on tumor tissue from most patients with-B cell acute lymphoblastic leukemia (B-ALL), non-Hodgkin's lymphoma (NHL), and chronic lymphocytic leukemia (CLL). CAR-T cells targeting CD19 have emerged to present a marked efficacy to directly eradicate liquid tumors and induce sustained tumor regression of B lineage cell malignancies (31, 32). It is worth noting that cytokines released by CD19 CAR-T cells also demonstrated the ability to activate both innate and adaptive immune systems and enhance tumor rejection.

Besides CD19-targeted CAR-T, CD22 and CD123 targeted CAR-T were also developed to treat leukemia. CD22-targeted CAR-T cells have a potent antileukemic activity and modest off-target toxicity (33). Since CD123 is expressed on a range of hematological malignancies, CAR-T targeting CD123 has also a potential role in the prevention of tumor progression and with additional therapeutic effects on eradicating the central nervous system in hematological malignancies (34). In general, all these different targeting CAR-T therapies provide rapid, efficient, and uninhibited regression and destruction of B-cell cancers.

### Chimeric Antigen Receptor-T Cell for Solid Tumors

Besides leukemia, CAR-T-cell therapy has been demonstrated to be effective against several kinds of solid tumors recently, including melanoma, colon cancer, non-small cell lung cancer, ovarian cancer, mesothelioma, and neuroblastoma (35). Mesothelin, epidermal growth factor receptor (EGFR), and human epidermal growth factor receptor 2 (HER2) are the most commonly focused antigen targets in CAR-T therapy to solid malignancies (Table 1).

**Table 1:** Engineering strategies for effector chimeric antigen receptor-T (CAR)-T cell.

**Specific antigens for engineered receptors**	**Treated cancer types**	**Function/effects**
CD19	B-NHL (DLBCL, follicular lymphoma, mantle cell lymphoma), CLL, B-ALL	Leukemia-specific targeting
CD22	B-ALL	
CD123	AML	
	B-ALL	
Mesothelin	Mesothelioma, TNBC, pancreatic cancer, lung cancer, gastric cancer, ovarian cancer, bile duct carcinoma	Cancer-specific targeting in solid tumor
EGFR/EGFRvIII	NSCLC, glioblastoma	
HER2	Ovarian cancer, breast cancer, osteosarcoma, HER2-positive sarcoma, glioblastoma	
CD133	Glioblastoma, lung cancer, breast cancer, liver cancer, gastric cancer, ovarian cancer, pancreatic cancer, colorectal cancer, prostate cancer	Cancer stem cell-specific targeting
CD90		
EpCAM		
ALDH		
PSCA	Prostate cancer	

*ALDH, aldehyde dehydrogenases; AML, acute myeloid leukemia; B-ALL, acute B lymphoblastic leukemia; B-NHL, B cell non-Hodgkin's lymphoma; CLL, chronic lymphocytic leukemia; DLBCL, diffuse large B-cell lymphoma; EGFR, epidermal growth factor receptor; EGFRvIII, the epidermal growth factor receptor variant III; EpCAM, epithelial cell adhesion molecule; HER2, human epidermal growth factor receptor 2; NSCLC, non-small-cell lung carcinoma; PSCA, prostate stem cell antigen; TNBC, triple-negative breast cancer*.

Mesothelin is a 40-kDa cell-surface glycoprotein. The precursor protein is proteolytically processed into two proteins, a 30-kDa soluble megakaryocyte potentiation factor and a 40-kDa GPI-anchored plasma membrane protein mesothelin (36). Mesothelin is limitedly expressed on mesothelial cells in different types of tissues including pleura, peritoneum, and pericardium but is highly expressed as a tumor-differentiation antigen in a broad spectrum of solid tumors, which make mesothelin as an attractive target for cancer immunotherapy (37). Mesothelin-specific CAR-T-cell therapy also has attracted widespread interest. Commonly, mesothelin-CAR-T-cells consist of anti-mesothelin scFv SS1 fused to TCRzeta signaling and costimulatory domains. There are various clinical studies to investigate the safety and feasibility of mesothelin-specific CAR-T in mesothelioma, lung cancer, breast cancer with pleural metastases (38). All of these trials demonstrated that the application of mesothelin-specific CAR-T-cell therapy is a promising strategy for mesothelin-expressing malignancies.

EGFR is a 170-kDa transmembrane glycoprotein belonging to the ErbB oncogene family of tyrosine kinase receptors. Aberrant activation of EGFR leads to autophosphorylation of receptor tyrosine kinase that ultimately drives cell proliferation and metastasis in various types of tumors (39). Anti-EGFR CAR-T therapy is considered as an alternative way for EGFR-overexpressed solid malignancies (40).

Human epidermal growth factor 2 (HER2) is a membrane tyrosine of the EGFR family. HER2 promotes oncogenesis of several cancers and is found to be amplified in multiple tumors. An αHER2/CD3 RNA-engineered CAR-T-like T cells have achieved success in treating HER2^+^ malignancies (41). Furthermore, HER2-specific CAR-T cells present a prominent effect in targeting and killing HER2-positive cancers such as GBM, ovarian cancer, and breast cancer (42). HER2-specific CAR-T cells along with PD1 blockade was demonstrated to have a significant therapeutic potential for glioblastoma (43). In addition to preclinical studies, clinical trials also revealed the safety, feasibility, and activity of CAR-T immunotherapy targeting HER2 in patients with advanced biliary tract cancers (BTCs) and pancreatic cancer (PCs) (44).

### Chimeric Antigen Receptor-T Cell Targeting Cancer Stem Cells

Cancer stem cells (CSCs) are a subpopulation of tumor cells that mimic self-renewal and multilineage differentiation capacity of normal tissues and are responsible for maintaining tumor heterogeneity, enhancing tumor growth, therapeutic resistance, immune evasion, invasion, and metastasis (45–47). Thus, specifically targeting CSCs is crucial for developing effective therapeutics. Numerous surface markers expressed on CSCs, namely, CD133, are applied to identify CSCs and thus provide potential targets for CAR-T cell therapy. A study demonstrated that anti-CD133 CAR-T cells efficiently eliminate glioblastoma stem cells *in vitro* and *in vivo* (48). Meanwhile, a case report indicated the safety and feasibility of the combination treatment of anti-EGFR CAR-T cells and anti-CD133 CAR-T cells in patients with cholangiocarcinoma (49). Besides CD133, prostate stem cell antigen (PSCA), CD90, EpCAM, and ALDH also can be considered as important target antigens for CAR-T-cell therapy in cancer treatment, which needs further study in both preclinical and clinical settings (50, 51).

### Challenges and Solutions of Chimeric Antigen Receptor-T Cell Therapy

#### Adverse Effects

Adverse effects are accompanied by all cancer therapies and sometimes can be a big challenge. The toxicities of CAR-T cell therapy include cytokine release syndrome (CRS), immune effector cell-associated neurotoxicity syndrome (ICANS), cytopenias, and B-cell aplasia (related to CD19 targeting CART cells). CRS and ICANS have emerged as dominant CAR-T-cell-mediated toxicities. The onset of the CRS correlates with T-cell activation and high levels of cytokines, which have no target preference as it can be observed in both CD19 and other novel CARs. The choice of costimulation domain can be a major predictor of toxicity. For 4-1BB incorporated CARs, there were 58% patients who had CRS of any grade at a median of 5 days from infusion (52). Compared with 4-1BB costimulation domain, incorporation of CD28 leads to more rapid and higher peak expansion of CAR-T cells (53), leading to a 93% incidence of any grade CRS at a median of 2 days from CAR-T-cell infusion (54). The severity of the CRS is also associated with tumor burden at the time of treatment (55), as heavy tumor burden can provide more stimulation for CAR-T cell expansion. The cause of ICANS remains poorly understood. Potential causes include direct central nervous system toxicity by the CAR-T cells, diffusion of inflammatory cytokines through the blood–brain barrier (BBB), and the dysfunction of the BBB caused by CAR-T cells and/or cytokines.

The safety of CAR-T-cell therapies can be improved by early drug intervention such as using glucocorticoids and tocilizumab, an anti-IL6 receptor antagonist, for CRS treatment, and optimal genetic engineering strategies to reduce CAR-T cell toxicity (56).

#### Low Efficacy in Solid Tumors

Unlike hematological malignancies, solid tumors present several barriers that affect the safety and clinical outcomes of CAR-T-cell therapy. Target antigen specificity and heterogeneity, lymphocyte trafficking, and tumor-induced immunosuppression are three major factors that hindered the efficacy of CAR-T immunotherapy in solid tumors. Types of approaches are currently explored to address these challenges to enhance treatment efficacy (Table 1).

Selection of an optimal tumor-associated antigen (TAA) is considered as one of the most significant steps for CAR-T targeting. TAA should be highly expressed on all tumor cells but hopefully not expressed on the normal tissues. Although various tumor antigens including neoantigens, oncofetal antigens, and tumor-selective antigens are investigated for CAR-T-cell therapy, there remain no ideal ones meeting the criteria of specificity (57). Another major limitation to TAAs of solid tumors is antigen heterogeneity, which is a variable of the expression of antigen on the cells within a given tumor. To date, employing more targets, targeting multiple tumor antigens at once, and exploring new antigen-activated T-cell killing pathways are three approaches to address the problem of tumor antigen specificity and heterogeneity (58).

Insufficient migration and infiltration to tumor sites is the additional challenge of CAR-T-cell treatment to solid tumors. It is demonstrated that persistence and intratumoral accumulation of CAR-T is inevitably limited after adoptive transfer especially in the liver, lung, and spleen (59). It could be partially attributed to downregulation of cellular adhesion molecules, which inhibits T-cell transmigration. Besides, solid tumors have the capacity of modifying the structure of the adjacent tissue that hinders intratumoral lymphocyte accumulation. Plenty of strategies have been applied to increase lymphocyte migrating and infiltrating to tumor sites (60). Local administration can enhance the accumulation of engineered CAR-T cells at tumor sites and superior control of tumor growth compared with systemic administration, as shown by several preclinical solid tumor models (61, 62). Chemokine receptor–ligand interactions plays an important role in mediating endogenous immune cell trafficking. CAR-T cells can be modified to express certain cytokine receptors to enhance trafficking into tumor tissue. For example, expression of a functional CCR2 receptor can enhance tumor localization and tumor eradication of the mesothelin-CAR-T cells (63).

Tumor immunosuppressive microenvironment hinders the efficacy of CAR-T therapy even if the T cell is successfully trafficking the tumor sites. The anatomical structure generated by tumor stroma and the associated high tissue pressure provide natural barriers for CAR-T therapy, while hypoxia and nutrient starvation are two factors of metabolic barriers (64). Limiting the numbers of tumor stroma cells, exposed to a hyperoxia environment, and manipulating key cellular regulators of nutrients, have shown the attractive outcomes to augment antitumor immunity and repress tumor growth (65, 66). Additionally, tumor-derived cytokines, namely, TGFβ, might reduce the antitumor response of CAR-T therapy (67). Theoretically, engineering novel CAR-T cells expressing negative TGFβ receptor might be beneficial. Furthermore, inhibitory leukocytes like regulatory T cells, tumor-associated macrophages (TAMs), and myeloid-derived suppressor cells (MDSCs) present as potent barriers of CAR-T therapy (68, 69). Reprogramming of the immunosuppressive nature of the TME by genetically engineering CAR-T cells with immune-modulating cytokines is the most commonly used strategy to address this problem, which should be explored in future studies.

#### Chimeric Antigen Receptor-T-Cell Therapy Resistance

CAR-T therapy resistance is also a challenge in the field. Various CD19 mutations and alternative splicing have been the dominant cause of CAR-T-cell resistance. In this setting, multivalent targeting CARs or serial manipulation with multiple different CAR-T cells may prevent single-agent resistance. The combination of CD19 and BCMA targeted CAR-T cells, either combined infusion of both anti-CD19 and anti-BCMA CAR-T cells or a tan-CAR with both a scFv-CD19 and scFv-BCMA in tandem orientation, may help to reduce the rate of relapse in the treatment with single scFv-CAR-T cells (70, 71). The same strategy can be explored for solid tumors.

Altogether, CAR-T cell therapy has proven to be an inspiring strategy for cancer treatment. Various studies have highlighted that CAR-T cells have achieved encouraging outcomes in various malignancies, while several barriers including the selection of TAA, lymphocyte trafficking, and tumor immunosuppression in solid tumors restrict the effects. There remains much for us to explore to enhance the therapeutic effects of CAR-T cells in cancer treatment (72).

## Therapeutic Strategies Targeting Bone Marrow-Derived Suppressor Cells

Bone marrow-derived suppressor cells (MDSCs) consist of a cluster of highly heterogeneous cells generated from myeloid progenitors, which protect a tumor from the immune system and restrain the efficacy of immunotherapy (73, 74). The immunosuppressive cytokines caused by tumor-related chronic inflammation induce normal myeloid cell precursors to proliferate and differentiate into MDSCs, which suppress the antitumor effect and promote tumor progression (75). Therefore, targeted numerous enzymes, growth factors, and cytokines regulating the lifecycle of MDSCs may serve as efficient ways to eliminate cancer (76). For instance, neutralizing antibody to KIT significantly reduced MDSC expansion and unleash anti-tumor efficacy of T cells in colon carcinoma (77). Antagonists of CXCR2 (S-265610) and CXCR4 (AMD3100) altered the recruitment of immature myeloid cells (iMCs) to the tumor and thus reverted the environment that favors tumor progression (78). Besides, anti-IL-6R mAb could eliminate the accumulation of MDSCs, subsequently upregulating IFNγ and enhancing antitumor T-cell response (79). In summary, eliminating MDSCs is a promising way to unleash immunosuppression in a tumor microenvironment and kill cancer.

## Therapeutic Strategies Targeting Tumor-Associated Macrophages

Tumor-associated macrophages (TAMs) are the most abundant MDSCs in the TME. They secrete various cytokines, growth factors, chemokines as well as inflammatory mediators that promote key processes in tumor progression (80–82). TAMs function in the processes of angiogenesis, invasion, and metastasis. TAM-induced immunosuppression is mediated by the expression of inhibitory checkpoints, including PD-L1, PD-L2, and the non-classical major histocompatibility complex (MHC) class I (MHC-I) molecules (83). Meanwhile, TAMs secrete several cytokines including IL-10, TGFβ, and CCL5, maintaining a strong immunosuppressive microenvironment by inducing regulatory T (T_reg_) cell expansion. TAMs also release arginase I to deplete L-arginine, which directly inhibits T-cell cytotoxicity (84).

TAMs exhibit roles of promoting tumor or inhibiting tumor upon different stimuli, which depend on the status of the polarization of macrophage (85). M1-like TAMs accumulating at very early phases of oncogenesis stimulate antitumor immunity and hold the capacity of tumoricidal effect. However, the persistence of M1-like TAMs can induce chronic inflammation, hence, enhancing genomic instability in tumor cells and acts as a driver of oncogenesis in the early oncogenesis (86). Various studies have demonstrated that M2-like TAMs present as the dominant subtype through the progress of “re-education” by contexture changes of immune cells and metabolic factors in TME (87, 88). With the characterization of plasticity and heterogeneity, reprogramming macrophage from M2- to M1-like may provide a viable strategy to eliminate tumor cells (89, 90). Accumulating researches are devoted to reversing the pro-tumor effect of TAMs (Table 2).

**Table 2:** *In vivo* or *in vitro* engineering strategies for macrophage-based immune therapy.

**Aims**	**Targets**	**Delivery strategy**	**Treated cancer type**	**Effects/function**
Recognition	CD47/SIRPα	*Ex vivo* and *in vivo* engineering	AML, pediatric brain cancer, B cell lymphoma, lung cancer, ovarian cancer	Enhancing phagocytosis
	CD40/CD40L	Activation *via* drug delivery (antibody, adenovirus vector)	Melanoma, mesothelioma, pancreatic ductal adenocarcinoma,	Activating antigen-presenting; substituting function of CD4+ T cells; apoptosis induction
Inhibition	CSF1/CSF1R	Drug delivery (antibody and small molecules)	Glioblastoma, tenosynovial giant cell tumors, Hodgkin lymphoma, colorectal cancer, fibrosarcoma, breast cancer	TAM depletion
	Phagocytosis	Delivery of bisphosphonates	Teratocarcinoma, rhabdomyosarcoma, breast cancer, lung cancer, melanoma, liver cancer, prostate cancer	
	CCL2/CCR2	Drug delivery (antibody and small molecules)	Ovarian cancer, lung cancer, melanoma, prostate cancer, liver cancer, breast cancer, pancreatic cancer	Blocking TAM recruitment
	IL-10	*In vivo* delivery of inhibitors	Lung cancer, ovarian cancer	Unleashing expression of IL-12
	DICER	NA	Lung cancer, colorectal cancer	Enhancing expression of IFNγ-STAT1 and repolarizing TAMs *via* miRNA biosynthesis inhibition
	HDACs	Delivery of TMP195	Breast cancer	Repolarizing TAMs, activating CCL1 and CCL2 expression *via* epigenetic remodeling
	LDHA	NA	Lung cancer	Downregulation of VEGF and PD-L1; reducing glycolysis and reversing TAM-driven immunosuppression
	PIK3γ	*In vivo* drug delivery (IPI-549)	Lung cancer, head and neck cancer	Reducing glycolysis and reversing TAM-driven immunosuppression; upregulation of MHC-II and IL-12; recruitment of antitumor immune cells
Activation	TLRs	Delivery of small molecules	Melanoma, breast cancer, ovarian cancer, lung cancer, head and neck cancer, renal cancer, endometrial cancer, cervical cancer, and types of leukemia	Activation of innate immune response

*AML, acute myeloid leukemia; CCL1, chemokine (C-C motif) ligand 1; CCL2, chemokine (C–C motif) ligand 2; CCR2, C–C chemokine receptor type 2; CSF1, colony-stimulating factor 1; CSF1R, colony-stimulating factor 1 receptor; HDACs, histone deacetylases; IFNγ, interferon gamma; IL-10, interleukin 10; IL-12, interleukin 12; LDHA, lactate dehydrogenase A; MHC-II, major histocompatibility complex class II; PD-L1, programmed death-ligand 1; PIK3γ, phosphoinositide 3-kinases gamma; SIRPα, signal regulatory protein α; STAT1, signal transducer and activator of transcription 1; TAM, tumor-associated macrophage; TLRs, Toll-like receptors; VEGF, vascular endothelial growth factor*.

Currently, depletion, recruitment inhibition, and reprogramming are three commonly used strategies of TAM targeting under clinical trial investigation (91). First, the most advanced approaches of TAM depletion depends on inhibition of colony-stimulating factor 1 and its receptor (CSF1/CSF1R) signaling. CSF1 binds with CSF1R, a class III receptor tyrosine kinase, regulating differentiation, migration, and survival of macrophage and its precursors (92). Various small molecules inhibiting CSF1R tyrosine kinase have been investigated in several researches. Preclinical researches revealed that PLX3397 inhibited tumor-associated microglia and enhances sensitivity to chemotherapy of glioma, c-kit-mutated melanoma, prostate cancer, and classical Hodgkin lymphoma (cHL) (93–95). In addition, CSF1R targeting small molecules, including ARRY-382, PLX7486, BLZ945, and JNJ-40346527, which target the intracellular tyrosine kinase of CSF1R, are in completed or ongoing studies in solid tumors and classical Hodgkin lymphoma (96). Antibodies also play an essential role in targeting CSF1/CSF1R. A study showed that a monoclonal antibody RG7155 strongly reduces TAMs with an increase in T-cell ratio in diffuse-type giant cell tumor patients (97). Furthermore, the compounds MCS110 and PD-0360324 targeting the ligand CSF1 are also found to have the capacity to effectively clear TAMs (95). Compared with the inhibitors of CSF1/CSF1R, bisphosphonates in liposomes seems to be a more directed approach of TAM depletion. Evidence was shown in lung cancer, melanoma, hepatocellular carcinoma, and lung metastasis from breast cancer that bisphosphonates significantly reduced TAM infiltration (98–100). However, there are plenty of side effects remaining in the strategy of TAM depletion. Anti-CSF1R antibodies also non-selectively target non-tumor macrophages with many safety concerns especially accompanied by complications. The increased expression of IFNγ and IFNα after CSF1R inhibition directly leads to upregulation of immune checkpoint molecules, such as PD-1 and CTLA-4, possibly restraining its therapeutic effects (101).

Second, blockade of monocyte recruitment to tumors serves as an alternative approach to hinder TAMs, namely, the application of CCL2/CCR2 inhibitors. CCR2, which is highly expressed in monocytes/TAMs, is the only known receptor for chemokine CCL2 (102). It is reported that employing CCR2 antagonist inhibits monocyte/TAM recruitment and M2 polarization in hepatocellular carcinoma (HCC) (102). Carlumab is the most representative CCL2-targeted antibody, which successfully represses macrophage infiltration and thus reduces tumor growth (103). Recently, PF-04136309, a small molecule targeting CCR2, was investigated in a clinical trial of pancreatic cancer, which could enhance sensitivity to chemotherapy (104). However, anti-CCL2/CCR2 therapy might have a notable side effect that the antitumor immune cells might be unable to target the tumors (105). Generally, both depletion and recruitment inhibition present inevitable toxicity and side effects.

Functional reprogramming of TAMs is an attractive way for cancer therapy and holds the capacity of providing an opportunity to rebalance the microenvironment immune infiltrate therapeutically from a pro-tumoral one to an antitumoral one (92, 106, 107). Anti-CD47 antibodies block the binding of CD47 to SIRPα, and thus increase phagocytosis of cancer cells, representing an efficient strategy of TAM reprogramming (83, 108). Inhibition of IL-10, a TAM-derived cytokine with the ability to block IL-12 and suppress T-cell tumoricidal function, was identified to improve the efficacy of chemotherapy (109). As Toll-like receptors (TLRs) act a critical role in innate immune response and polarize macrophages into a pro-inflammatory subtype, studies investigate different ligands to change the subtype of TAMs to an antitumoral one (110). The results indicated that agonists of TLR7, TLR8, and TLR9 induced macrophage repolarization and increased tumoricidal activity in several cancers (111–113). Additionally, CD40, a receptor commonly expressed in antigen-presenting cells, interacts with CD40L expressed by T cells to increase pro-inflammatory cytokines. Agonists of CD40/CD40L were identified to affect the protumoral effects in several cancers. Furthermore, strategies targeting crucial processes especially epigenetic regulation in gene expression also obtain effective outcomes to reprogramming TAMs (114). Studies revealed that inhibition of DICER, a key enzyme for microRNA synthesis in macrophages switches the subtype accompanied by tumor regression and infiltration of effective immune cells (115). Inhibitors of histone deacetylase (HDACs) can repolarize the phenotype of TAMs and alter CCL1 expression in monocytes (116). Besides, metabolic reprogramming also plays a significant role in functional modifying of TAMs. Deletion of LDHA and inhibition of phosphatidylinositol-4, 5-bisphosphate 3-kinase catalytic subunit gamma (PIK3γ) aimed to reduce glycolysis and hence relieved TAM-driven immunosuppression (117, 118). What is more, lactic acid produced by aerobic or anaerobic glycolysis has an essential function in inducing M2-like polarization of TAMs, suggesting the possibilities to reprogram TAMs by suppressing the production of lactic acid (119). However, TAM reprogramming *via* depleting M2-like TAMs and/or favoring their repolarization toward an M1-like phenotype is limited by innate and acquired resistance, compensation by alternative immunosuppressive cells, and relapse during treatment discontinuation. Besides, side effects including anemia and autoimmunity disease are hard to overcome.

Similar to CAR-T-cell therapy, a recent study engineered the macrophage to express CARs (CAR-Ms) to have antigen-specific phagocytosis capacity, which induced a pro-inflammatory tumor environment, enhanced antitumor T-cell activity, and alleviated tumor burden (120). Another study generated induced pluripotent stem cell (iPSC)-derived, CAR-expressing macrophage cells (CAR-iMac) with antigen-dependent macrophage function and antitumor effects both *in vitro* and *in vivo* (121). In general, these studies provided potent strategies for reprogramming tumor microenvironment and set good examples reverting immunosuppression for cancer immunotherapy, though the persistence and efficacy of the CAR macrophage may be further modified.

## Therapeutic Strategies Targeting Regulatory T Cells

Regulatory T (Treg) cells suppress abnormal/excessive immune responses to self- and non-self-antigens to prevent chronic inflammatory, allergic, and autoimmune diseases and maintain immune homeostasis (122, 123). Infiltration of Treg cells into the TME occurs in various murine and human tumors (124). Treg-induced immune homeostasis can significantly limit the efficacy of antitumor effect as many tumor antigens are either overexpressed or mutated self-antigens. Notably, increasing expression of Tregs regulator forkhead box protein 3 (FOXP3) is identified in many tumors such as breast cancer, melanoma, and pancreatic cancer with a complicated function and cell type-related manner (125). It is reported that FOXP3 plays two key roles: (1) the tumor suppressor in prostate, ovarian, and breast cancers *via* activating tumor-suppressor genes and inhibiting several oncogenes; (2) a biomarker related with poor prognosis in melanoma, non-small cell lung cancer, urinary bladder cancer, and esophageal cancer (126). Besides, FOXP3 is also involved in immune functions of Tregs *via* inhibition of APC function mediated by CTLA-4, an increase in immunosuppressive cytokines and metabolites, IL-2 exhaustion (127). There are mainly three strategies to reprogram Treg function based on the immune-suppressive mechanisms of Treg cells (Table 3).

**Table 3:** Therapeutic strategies targeting regulatory T cell (Treg) for cancer therapy.

**Targets**	**Treated cancer type**	**Effects/function**
CD28^*^	NA	Inhibiting stability and function of Treg
CD25^*^	Breast cancer	Depleting Treg
CTLA4^*^	Melanoma, colorectal cancer, fibrosarcoma	Depleting CTLA4 expressing Treg through ADCC
GITR^#^	Bladder cancer, sarcoma, melanoma, lung cancer	Inhibiting the suppressive activity of Tregs; activating effector T cells
OX40^#^	Glioma, breast cancer, colon carcinoma, prostate cancer, sarcoma, melanoma, lung cancer	
CCL22/CCR4^*^	Lung cancer, esophageal cancer	Attenuating Treg accumulation
CCL28/CCR10^*^	Ovarian cancer	
CCL1/CCR8^*^	Breast cancer	
Nrp1^*^	Melanoma, CLL, cervical cancer	Preventing Treg recruitment; downregulating VEGF, and producing IFNγ
Akt-mTOR	Melanoma, ovarian cancer	Increasing glucose uptake and glycolysis; destabilizing Treg
TLR1^#^	AML, metastatic colorectal cancer, mantle cell lymphoma	Enhancing Treg glycolysis and proliferation
TLR2^#^	Melanoma, AML, metastatic colorectal cancer, mantle cell lymphoma	
TLR8^#^	Melanoma	Inhibiting glucose uptake and glycolysis
HIF1α^*^	Metastatic melanoma in lungs	Impairing Treg stability and driving Foxp3 degradation
HAT^*^	Breast cancer, prostate cancer, pancreatic cancer, ovarian cancer	Epigenetical inhibition of Foxp3
TET^*^		
Foxp3^*^		Inhibiting function of Tregs
CBM complex^*^	Melanoma, colorectal cancer	Enhancing IFNγ and suppressing tumor growth
Eos^*^	Lung cancer	Reprogramming Treg to gain immune-stimulating capacity; decreasing expression of Foxp3
Helios^*^	Melanoma, colorectal cancer	Decreasing expression of Foxp3
Foxo1/Foxo3^*^		
EZH2^*^		

* *Inhibition via drug delivery, decoys, siRNA, and others*.

# *Activation via mimics or ligands*.

*ADCC, antibody-dependent cellular cytotoxicity; Akt, protein kinase B; AML, acute myeloid leukemia; CBM complex, CARMA1–BCL10–MALT1 signalosome complex; CCL, chemokine (C-C motif) ligand; CCR, C-C chemokine receptor; CLL, chronic lymphocytic leukemia; CTLA4, cytotoxic T-lymphocyte-associated protein 4; EZH2, enhancer of zeste homolog 2; Foxo1, forkhead box O1; Foxo3, forkhead box O3; Foxp3, forkhead box P3; GITR, glucocorticoid-induced tumor necrosis factor receptor; HAT, histone acetyltransferase; HIF1α, hypoxia-inducible factor 1-alpha; IFNγ, interferon gamma; mTOR, mammalian target of rapamycin; Nrp1, neuropilin 1; TET, Ten-eleven translocation methylcytosine dioxygenase; TLRs, toll-like receptors; VEGF, vascular endothelial growth factor*.

High expression of several cell surface receptors makes them attractive targets to selectively deplete Treg cells. Interruption of costimulatory molecule CD28 in Tregs impairs their differentiation and function selectively within tumors, reducing their capacity to suppress antitumor immune responses and promoting tumor control (128). Similarly, targeting surface receptor CD25 successfully represses Treg cells with antitumor immune response (129–131). Moreover, since cytotoxic T-lymphocyte antigen-4 (CTLA4) is expressed by Treg cells and increased after activation of effective T cells, mAbs targeting CTLA4 was applied to antagonize inhibitory signal and activate effective T cells to regain tumoricidal activity (132). Other antibodies against GITR, OX40, and molecules predominantly expressed by Treg cells have long been used to selectively deplete Tregs and inhibit their suppressive capacity (133, 134). Additionally, blocking chemokine and chemokine receptors (CCL22-CCR4, CCL28-CCR10, and CCL1-CCR8) associated with Treg chemotaxis into TME could reduce the number of Tregs and increase antitumor immune responses (135–137). In murine cancer models, deletion of neuropilin 1 (Nrp-1) specifically in Tregs leads to enhanced immunity to many transplantable tumors (138).

Reprogramming metabolic profiles including glycolysis and lipid oxidation has been considered as another strategy to suppress Treg cells and change the fate of immunotherapy. Recent studies suggest that metabolic regulations are actively involved in Treg differentiation, Foxp3 expression, and Treg stability. Studies showed that inhibiting Akt–mTOR may regulate metabolic programs to facilitate the suppression of Treg cells (139). Besides, low oxygen tension combined with TCR activation, can stabilize hypoxia-inducible factor 1a (HIF1a) and promote Foxp3 expression (140). TLR1 and TLR2 signaling activation in Treg cells enhances Treg glycolysis and proliferation and unleash the immunosuppressive capacity (141). TLR8 signaling selectively inhibits glucose uptake and glycolysis in human Treg cells, resulting in reversal of Treg suppression in melanoma (142).

Modulation of critical factors and chromatin regulators associated with transcription can also transform the function of Treg cells. Foxp3 serves as the most significant transcription factor in Tregs, which is involved in the differentiation and stability of Tregs. Loss of Foxp3 results in autoimmunity in the normal situation, while deficiency of Foxp3 unleashes immunosuppressive capacities and, hence, improves tumoricidal activities (143, 144). In addition, epigenetical inhibition of Foxp3 *via* interruption of histone acetylation [histone acetyltransferase (HAT) EP300] and DNA methylation [ten-eleven translocation (TET)] reduces the immunosuppressive function of Tregs and leaves effective T cells to regain their antitumor function (145, 146). Foxp3 also regulates Tregs by interacting with other transcription factors; disruption of these factors provides an alternative way to reprogram Tregs with antitumor ability. It is reported that the disruption of the CARMA1–BCL10–MALT1 (CBM) signalosome complex and induction of IFNγ secretion suppress Tregs, activate the adaptive immune response, hinder tumor growth, and improve the efficacy of immune checkpoint therapy (147). Moreover, genetic or pharmacologic disruption of transcription factors Eos, Helios, Foxo1/Foxo3, and EZH2 reprograms Tregs to enhance cancer immunity and improve tumoricidal activity (148–151). However, while there is some success through the treatment of targeted Tregs, there are still some obstacles that need to be addressed. First, specific targets for reprogramming Tregs are limited, especially for tumor-infiltrating Tregs. Second, while an immune-related adverse effect resulting from systemic depletion of Treg cells becomes a risk for patients, strategies specifically tuning Treg cell function in TME are needed.

## Therapeutic Strategies Targeting Natural Killer Cells

Natural killer (NK) cells, which present in the peripheral blood, lymph nodes, spleen, and bone marrow, are innate immune cells involved in cytotoxicity and cytokine production (152). Tumor necrosis factor alpha (TNF-α), granulocyte–macrophage colony-stimulating factor (GM-CSF), and IFN-γ are the main cytokines activated by NK cells (153). In addition, a complicated network of activating and inhibitory receptors regulates the function of NK cells. C-type lectin receptors (CD94/NKG2C, NKG2D), killer cell C-type lectin-like receptor (NKp65, NKp80), natural cytotoxicity receptors (NKp30, NKp44, and NKp46), SLAM family receptors (2B4, SLAM6, and SLAM7, function in the recognition of hematopoietic cells), Fc receptor FcγR (function in antibody-dependent cell cytotoxicity), killer cell immunoglobulin-like receptors (KIR) (KIR-2DS and KIR-3DS), DNAM-1, and CD137 (41BB) serve as activating receptors, while KIR-2DL and C-type lectin receptors CD94/NKG2A/B serve as inhibitory receptors of NK cells (154, 155). NK cells play an important role in initiating and promoting cancer with effective capacity at the first-line defense for tumor elimination. The major functions of NK include cytotoxicity and cytokine production, which help in killing tumor cells. Higher infiltration of NK cells usually associates with a good prognosis in various cancers. However, due to the limited ability of homing and immunosuppressive tumor microenvironment, solid cancers commonly present a poor NK cell infiltration with increasing inhibitory signals. Therefore, targeting with an inhibition signal may serve as a meaningful approach to restore cytotoxic function of NK cells against cancer cells (154, 156, 157).

To date, NK-cell-based immunotherapy is roughly divided into two types: directly targeting cytokines and receptors involved in NK cell proliferation and function; and chimeric antigen receptor (CAR)-engineered NK cells (Table 4). IL-2 and IL-15 are two of the most commonly employed cytokines in targeting NK cells. IL-2 was applied to produce lymphokine-activated killer (LAK) cells with unsatisfactory outcomes, which is probably attributed to the expansion of Treg cells at the same time. Compared with IL-2, IL-15 met a great success in targeting NK cells for tumor treatment. Expansion of NK cells and CD8 effector memory T cells after IL-15 therapy was identified in both mouse model and clinical studies (158, 159). Other cytokines, including IL-18 and IL-21, have also been shown to promote NK cell functions (158, 160). What is more, it is worth concerning that a combination therapy of cytokine and other traditional therapy can elevate NK cell proliferation, cytotoxicity, and memory, which is more effective than single cytokine treatment. Additionally, antibodies targeting activating receptors involved in antibody-dependent cell-mediated cytotoxicity (ADCC) can also improve cytotoxicity of NK cells to tumor cells. Similarly, blocking of inhibitory receptors like KIR reverse the suppressive state of NK cells (161).

**Table 4:** Natural killer (NK) cell-based immune therapy.

**Targeted genes**	**Delivery strategy**	**Treated cancer type**	**Effects/function**
IL-2^*^	Delivery of superkine or fusion protein	AML	Promoting NK cell proliferation and activating NK cells
IL-15^*^	Delivery of fusion protein	Ovarian cancer, myeloid leukemia	Enhancing cytotoxicity of NK cells
IL-18^*^	Delivery of cIAP2 and TRAF1	Triple-negative breast cancer, lung cancer, melanoma	Sustaining NK cell survival
IL-21^*^	Delivery of rIL-21	Pancreatic cancer, mantle cell lymphoma, melanoma	
NKG2D^*^	Inhibition *via* antibody	Lung cancer, colon cancer, prostate cancer, ovarian cancer, CLL	Triggering cytokine production and NK cell cytotoxicity
CD19^#^	*Ex vivo* engineering NK	AML, ALL, multiple myeloma	Specific targeting *via* the CAR engineered
CD20^#^			
HER2^#^		Neuroblastoma, ovarian cancer, colon cancer, renal cell cancer, osteosarcoma	
EpCAM^#^			
GD2^#^			
PSCA^#^		Prostate cancer	

* *Inhibition via drug delivery, decoys, siRNA, and others*.

# *Recognition targets for ex vivo engineering*.

*NKG2D, natural-killer group 2, member D. ALL, acute lymphoblastic leukemia; AML, acute myeloid leukemia; CARs, chimeric antigen receptors; CLL, chronic lymphoblastic leukemia; EpCAM, epithelial cell adhesion molecule; HER2, human epidermal growth factor receptor 2; IL, interleukin; PSCA, prostate stem cell antigen*.

CAR-NK cell therapy has largely been investigated. CAR-NK cell therapy exhibits enhanced tumoricidal capacity with advantages that are not responsible for GVHD and do not induce cytokine storms (162). In addition, the sources of CAR-NK cells can be generated from cord blood (CB), peripheral blood (PB), adult hematopoietic stem cells (HSCs), embryonic stem cells (ESCs), and induced pluripotent stem cells (iPSCs) (163, 164). Similar as in CAR-T cells, an intracellular signaling domain-like CD3ζ and costimulatory signaling domain (CD28, 4-1BB) are basic structures for a CAR. Other molecules like DNAX-activation protein 12 (DAP12), DAP10, and NKG2D can also be selected as intracellular or ectodomain (162). For antigen selection, most CAR-NK cells target CD19 and CD20 in hematological malignancies, and HER2, EpCAM, GD2, and PSCA in solid cancers. Recent studies suggested that the most effective responses of CAR-NK cells were observed in ALL, prostate carcinoma, and osteosarcoma, while the effects in other cancers tested were not that satisfactory (165–167).

To sum up, the development of NK cell-based cancer immunotherapy is a fast-evolving field. Unleashing NK cell antitumor responses by harnessing surface receptors and involved cytokines depict potentially successful immunotherapeutic strategies for cancer. The foremost challenge of CAR-NK cell therapy is expansion of primary NK cells *ex vivo*. Additionally, limited transfection efficacy in NK cells to express CARs is also notable. Selection of a suitable method, such as viral infection, electroporation, and nanoparticles, is a prerequisite for successful CAR-NK therapy. Although there remain pressing obstacles of CAR-NK cells, the striking outcomes in several cancers make it a promising new strategy for cancer immunotherapy.

## Conclusions and Perspectives

**Figure 1:**
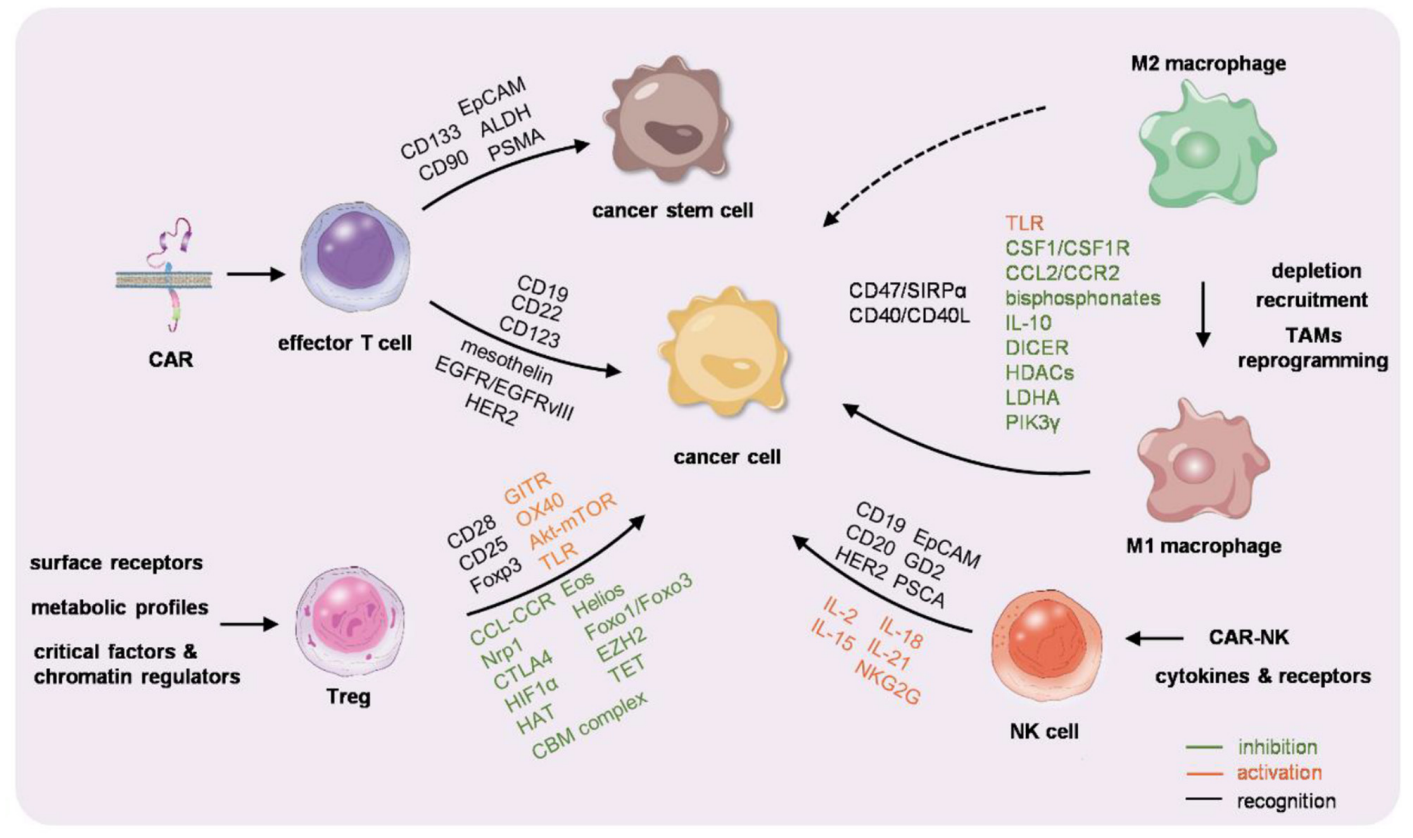
Graphic summarization of the immunocyte-based cancer therapy. Chimeric antigen receptor-T (CAR-T) cells, macrophages, regulatory T cells (Tregs), and natural killer (NK) cells are engineered or educated either *ex vivo* or *in vivo* to reactivate the immunity against cancer.

With the advances in the knowledge in the cross-talk between different immune cells and tumor cells, the techniques in cell engineering and drug delivery, and immunotherapies targeting accessory immune cells either *ex vivo* or *in vivo* have been intensively studied (Figure 1). Some of them have been widely used in clinics; some have been already under phase 2/3 clinical trial. Generally, immunotherapy is emerging as a promising strategy against a variety of cancers and might be the final therapeutic tool. Translational researches using different strategies for various types of cancers are intensive studies worldwide. Future challenges rely on improvement of the safety, efficacy, and convenience in personalization and customization.

## Author Contributions

YD, GY, and LL designed the paper. YD, ZW, XG, GY, and LL analyzed the references and edited the paper. YD and GY wrote the paper. All authors contributed to the article and approved the submitted version.

## Conflict of Interest

The authors declare that the research was conducted in the absence of any commercial or financial relationships that could be construed as a potential conflict of interest.

## References

[1] Chen W Zheng R Baade PD Zhang S Zeng H Bray F. Cancer statistics in China, 2015. CA Cancer J Clin. (2016) 66:115–32. 10.3322/caac.21338 26808342

[2] Torre LA Bray F Siegel RL Ferlay J Lortet-Tieulent J Jemal A. Global cancer statistics, 2012. CA Cancer J Clin. (2015) 65:87–108. 10.3322/caac.21262 25651787

[3] Kirkwood JM Butterfield LH Tarhini AA Zarour H Kalinski P Ferrone S. Immunotherapy of cancer in 2012. CA Cancer J Clin. (2012) 62:309–5. 10.3322/caac.20132 22576456 PMC3445708

[4] Gu L Mooney DJ. Biomaterials and emerging anticancer therapeutics: engineering the microenvironment. Nat Rev Cancer. (2016) 16:56–66. 10.1038/nrc.2015.3 26694936 PMC4790726

[5] Tang H Qiao J Fu YX. Immunotherapy and tumor microenvironment. Cancer Lett. (2016) 370:85–90. 10.1016/j.canlet.2015.10.009 26477683 PMC4725050

[6] Lei X Lei Y Li JK Du WX Li RG Yang J. Immune cells within the tumor microenvironment: biological functions and roles in cancer immunotherapy. Cancer Lett. (2020) 470:126–33. 10.1016/j.canlet.2019.11.009 31730903

[7] Dominguez-Andres J Netea MG. Long-term reprogramming of the innate immune system. J Leukoc Biol. (2019) 105:329–38. 10.1002/JLB.MR0318-104R 29999546

[8] Gonzalez H Hagerling C Werb Z. Roles of the immune system in cancer: from tumor initiation to metastatic progression. Genes Dev. (2018) 32:1267–84. 10.1101/gad.314617.118 30275043 PMC6169832

[9] Vinay DS Ryan EP Pawelec G Talib WH Stagg J Elkord E. Immune evasion in cancer: mechanistic basis and therapeutic strategies. Sem Cancer Biol. (2015) 35:S185–98. 10.1016/j.semcancer.2015.03.004 25818339

[10] Ribas A Adaptive immune resistance: how cancer protects from immune attack. Cancer Discov. (2015) 5:915–9. 10.1158/2159-8290.CD-15-0563 26272491 PMC4560619

[11] Lanza R Russell DW Nagy A. Engineering universal cells that evade immune detection. Nat Rev Immunol. (2019) 19:723–33. 10.1038/s41577-019-0200-1 31417198

[12] Wang H Mooney DJ. Biomaterial-assisted targeted modulation of immune cells in cancer treatment. Nat Mat. (2018) 17:761–72. 10.1038/s41563-018-0147-9 30104668

[13] Bantug GR Galluzzi L Kroemer G Hess C. The spectrum of T cell metabolism in health and disease. Nat Rev Immunol. (2018) 18:19–34. 10.1038/nri.2017.99 28944771

[14] Lim WA June CH. The principles of engineering immune cells to treat cancer. Cell. (2017) 168:724–40. 10.1016/j.cell.2017.01.016 28187291 PMC5553442

[15] Schumacher TN Schreiber RD. Neoantigens in cancer immunotherapy. Science (New York, NY). (2015) 348:69–74. 10.1126/science.aaa4971 25838375

[16] Yarchoan M Johnson BA III Lutz ER Laheru DA Jaffee EM. Targeting neoantigens to augment antitumour immunity. Nat Rev Cancer. (2017) 17:209–22. 10.1038/nrc.2016.154 28233802 PMC5575801

[17] Robbins PF. Tumor-infiltrating lymphocyte therapy and neoantigens. Cancer J (Sudbury, Mass). (2017) 23:138–43. 10.1097/PPO.0000000000000267 28410302

[18] Rowshanravan B Halliday N Sansom DM. CTLA-4: a moving target in immunotherapy. Blood. (2018) 131:58–67. 10.1182/blood-2017-06-741033 29118008 PMC6317697

[19] Constantinidou A Alifieris C Trafalis DT. Targeting programmed cell death−1 (PD-1) and ligand (PD-L1): a new era in cancer active immunotherapy. Pharmaco Ther. (2018) 194:84–106. 10.1016/j.pharmthera.2018.09.008 30268773

[20] Zhang J Dang F Ren J Wei W. Biochemical aspects of PD-L1 regulation in cancer immunotherapy. Trends Biochem Sci. (2018) 12:1014–32. 10.1016/j.tibs.2018.09.004 30287140 PMC6252278

[21] Gomes-Silva D Ramos CA. Cancer immunotherapy using car-T cells: from the research bench to the assembly line. Biotechnol J. (2018) 13:1700097. 10.1002/biot.201700097 28960810 PMC5966018

[22] Rahbarizadeh F Ahmadvand D Moghimi SM. CAR T-cell bioengineering: single variable domain of heavy chain antibody targeted CARs. Adv Drug Deliv Rev. (2019) 141:41–6. 10.1016/j.addr.2019.04.006 31004624

[23] Liu J Zhong JF Zhang X Zhang C. Allogeneic CD19-CAR-T cell infusion after allogeneic hematopoietic stem cell transplantation in B cell malignancies. J Hematol Oncol. (2017) 10:35. 10.1186/s13045-017-0405-3 28143567 PMC5282795

[24] Ramos CA Dotti G. Chimeric antigen receptor (CAR)-engineered lymphocytes for cancer therapy. Expert Opin Biol Ther. (2011) 11:855–73. 10.1517/14712598.2011.573476 21463133 PMC3107373

[25] Getts D Hofmeister R Quintas-Cardama A. Synthetic T cell receptor-based lymphocytes for cancer therapy. Adv Drug Deliv Rev. (2019) 141:47–54. 10.1016/j.addr.2019.04.002 30981835

[26] Maher J Wilkie S Davies DM Arif S Picco G Julien S. Targeting of tumor-associated glycoforms of MUC1 with CAR T cells. Immunity. (2016) 45:945–6. 10.1016/j.immuni.2016.10.014 27851917

[27] Chmielewski M Hombach AA Abken H. Of CARs and TRUCKs: chimeric antigen receptor (CAR) T cells engineered with an inducible cytokine to modulate the tumor stroma. Immunol Rev. (2014) 257:83–90. 10.1111/imr.12125 24329791

[28] Brocker T Karjalainen K. Signals through T cell receptor-zeta chain alone are insufficient to prime resting T lymphocytes. J Exp Med. (1995) 181:1653–9. 10.1084/jem.181.5.1653 7722445 PMC2192006

[29] Cox DBT Gootenberg JS Abudayyeh OO Franklin B Kellner MJ Joung J. RNA editing with CRISPR-Cas13. Science (New York, NY). (2017) 358:1019–027. 10.1126/science.aaq0180 PMC5793859 29070703

[30] Sha H-h Wang D-d Yan D-l Hu Y Yang S-j Liu S-w. Chimaeric antigen receptor T-cell therapy for tumour immunotherapy. Biosci Rep. (2017) 37:BSR20160332. 10.1042/BSR20160332 28053197 PMC5270315

[31] Sadelain M. CD19 CAR T Cells. Cell. (2017) 171:1471. 10.1016/j.cell.2017.12.002 29245005

[32] Li X Ding Y Zi M Sun L Zhang W Chen S. CD19, from bench to bedside. Immunol Lett. (2017) 183:86–95. 10.1016/j.imlet.2017.01.010 28153605

[33] Fry TJ Shah NN Orentas RJ Stetler-Stevenson M Yuan CM Ramakrishna S. CD22-targeted CAR T cells induce remission in B-ALL that is naive or resistant to CD19-targeted CAR immunotherapy. Nat Med. (2018) 24:20–8. 10.1038/nm.4441 29155426 PMC5774642

[34] Testa U Pelosi E Castelli G. CD123 as a therapeutic target in the treatment of hematological malignancies. Cancers. (2019) 11:1358. 10.3390/cancers11091358 31547472 PMC6769702

[35] Guo Y Wang Y Han W. Chimeric antigen receptor-modified T cells for solid tumors: challenges and prospects. J Immunol Res. (2016) 2016:3850839. 10.1155/2016/3850839 26998495 PMC4779545

[36] Robinson BW Creaney J Lake R Nowak A Musk AW de Klerk N. Mesothelin-family proteins and diagnosis of mesothelioma. Lancet (London, England). (2003) 362:1612–6. 10.1016/S0140-6736(03)14794-0 14630441

[37] Morello A Sadelain M Adusumilli PS. Mesothelin-targeted CARs: driving T cells to solid tumors. Cancer Discov. (2016) 6:133–46. 10.1158/2159-8290.CD-15-0583 26503962 PMC4744527

[38] O'Hara M Stashwick C Haas AR Tanyi JL. Mesothelin as a target for chimeric antigen receptor-modified T cells as anticancer therapy. Immunotherapy. (2016) 8:449–60. 10.2217/imt.16.4 26973126 PMC5619020

[39] Tebbutt N Pedersen MW Johns TG. Targeting the ERBB family in cancer: couples therapy. Nat Rev Cancer. (2013) 13:663–73. 10.1038/nrc3559 23949426

[40] Janku F Stewart DJ Kurzrock R. Targeted therapy in non-small-cell lung cancer–is it becoming a reality? Nat rev Clin Oncol. (2010) 7:401–14. 10.1038/nrclinonc.2010.64 20551945

[41] Luo F Qian J Yang J Deng Y Zheng X Liu J. Bifunctional alphaHER2/CD3 RNA-engineered CART-like human T cells specifically eliminate HER2(+) gastric cancer. Cell Res. (2016) 26:850–3. 10.1038/cr.2016.81 27339087 PMC5129888

[42] Yu S Li A Liu Q Li T Yuan X Han X. Chimeric antigen receptor T cells: a novel therapy for solid tumors. J Hematol Oncol. (2017) 10:78. 10.1186/s13045-017-0444-9 28356156 PMC5372296

[43] Abels ER Maas SL Nieland L Wei Z Cheah PS Tai E. Glioblastoma-associated microglia reprogramming is mediated by functional transfer of extracellular miR-21. Cell Rep. (2019) 28:3105–19.e7. 10.1016/j.celrep.2019.08.036 31533034 PMC6817978

[44] Feng K Liu Y Guo Y Qiu J Wu Z Dai H. Phase I study of chimeric antigen receptor modified T cells in treating HER2-positive advanced biliary tract cancers and pancreatic cancers. Protein Cell. (2018) 9:838–47. 10.1007/s13238-017-0440-4 28710747 PMC6160389

[45] Eun K Ham SW Kim H. Cancer stem cell heterogeneity: origin and new perspectives on CSC targeting. BMB Rep. (2017) 50:117–25. 10.5483/BMBRep.2017.50.3.222 27998397 PMC5422023

[46] Prager BC Xie Q Bao S Rich JN. Cancer stem cells: the architects of the tumor ecosystem. Cell Stem Cell. (2019) 24:41–53. 10.1016/j.stem.2018.12.009 30609398 PMC6350931

[47] Wang T Shigdar S Gantier MP Hou Y Wang L Li Y. Cancer stem cell targeted therapy: progress amid controversies. Oncotarget. (2015) 6:44191–206. 10.18632/oncotarget.6176 26496035 PMC4792551

[48] Gopisetty G Xu J Sampath D Colman H Puduvalli VK. Epigenetic regulation of CD133/PROM1 expression in glioma stem cells by Sp1/myc and promoter methylation. Oncogene. (2013) 32:3119–29. 10.1038/onc.2012.331 22945648 PMC3820114

[49] Feng KC Guo YL Liu Y Dai HR Wang Y Lv HY. Cocktail treatment with EGFR-specific and CD133-specific chimeric antigen receptor-modified T cells in a patient with advanced cholangiocarcinoma. J Hematol Oncol. (2017) 10:4. 10.1186/s13045-016-0378-7 28057014 PMC5217546

[50] Guo Y Feng K Wang Y Han W. Targeting cancer stem cells by using chimeric antigen receptor-modified T cells: a potential and curable approach for cancer treatment. Protein Cell. (2018) 9:516–26. 10.1007/s13238-017-0394-6 28290053 PMC5966354

[51] Yu H Pan J Guo Z Yang C Mao L. CART cell therapy for prostate cancer: status and promise. OncoTargets Ther. (2019) 12:391–5. 10.2147/OTT.S185556 30655675 PMC6322708

[52] Schuster SJ Bishop MR Tam CS Waller EK Borchmann P McGuirk JP. Tisagenlecleucel in adult relapsed or refractory diffuse large B-cell lymphoma. N Engl J Med. (2019) 380:45–56. 10.1056/NEJMoa1804980 30501490

[53] Neelapu SS Locke FL Bartlett NL Lekakis LJ Miklos DB Jacobson CA. Axicabtagene ciloleucel CAR T-cell therapy in refractory large B-cell lymphoma. N Engl J Med. (2017) 377:2531–44. 10.1056/NEJMoa1707447 29226797 PMC5882485

[54] Lee DW Gardner R Porter DL Louis CU Ahmed N Jensen M. Current concepts in the diagnosis and management of cytokine release syndrome. Blood. (2014) 124:188–95. 10.1182/blood-2014-05-552729 24876563 PMC4093680

[55] Davila ML Riviere I Wang X Bartido S Park J Curran K. Efficacy and toxicity management of 19-28z CAR T cell therapy in B cell acute lymphoblastic leukemia. Sci Transl Med. (2014) 6:224ra25. 10.1126/scitranslmed.3008226 24553386 PMC4684949

[56] Perales MA Kebriaei P Kean LS Sadelain M. Reprint of: building a safer and faster CAR: seatbelts, airbags, and CRISPR. Biol Blood Marrow Transplant J Am Soc Blood Marrow Transplant. (2018) 24:S15–9. 10.1016/j.bbmt.2017.12.789 29425516

[57] Restifo NP Dudley ME Rosenberg SA. Adoptive immunotherapy for cancer: harnessing the T cell response. Nat Rev Immunol. (2012) 12:269–81. 10.1038/nri3191 22437939 PMC6292222

[58] Sampson JH Choi BD Sanchez-Perez L Suryadevara CM Snyder DJ Flores CT. EGFRvIII mCAR-modified T-cell therapy cures mice with established intracerebral glioma and generates host immunity against tumor-antigen loss. Clin Cancer Res. (2014) 20:972–84. 10.1158/1078-0432.CCR-13-0709 24352643 PMC3943170

[59] Harlin H Meng Y Peterson AC Zha Y Tretiakova M Slingluff C. Chemokine expression in melanoma metastases associated with CD8^+^ T-cell recruitment. Cancer Res. (2009) 69:3077–85. 10.1158/0008-5472.CAN-08-2281 19293190 PMC3886718

[60] Craddock JA Lu A Bear A Pule M Brenner MK Rooney CM. Enhanced tumor trafficking of GD2 chimeric antigen receptor T cells by expression of the chemokine receptor CCR2b. J Immunother (Hagerstown, Md.: 1997). (2010) 33:780–8. 10.1097/CJI.0b013e3181ee6675 20842059 PMC2998197

[61] Adusumilli PS Cherkassky L Villena-Vargas J Colovos C Servais E Plotkin J. Regional delivery of mesothelin-targeted CAR T cell therapy generates potent and long-lasting CD4-dependent tumor immunity. Sci Transl Med. (2014) 6:261ra151. 10.1126/scitranslmed.3010162 25378643 PMC4373413

[62] Parente-Pereira AC Burnet J Ellison D Foster J Davies DM van der Stegen S. Trafficking of CAR-engineered human T cells following regional or systemic adoptive transfer in SCID beige mice. J Clin Immunol. (2011) 31:710–8. 10.1007/s10875-011-9532-8 21505816

[63] Moon EK Carpenito C Sun J Wang LC Kapoor V Predina J. Expression of a functional CCR2 receptor enhances tumor localization and tumor eradication by retargeted human T cells expressing a mesothelin-specific chimeric antibody receptor. Clin Cancer Res. (2011) 17:4719–30. 10.1158/1078-0432.CCR-11-0351 21610146 PMC3612507

[64] Zhang Y Ertl HC. Starved and asphyxiated: how can CD8(+) T cells within a tumor microenvironment prevent tumor progression. Front Immunol. (2016) 7:32. 10.3389/fimmu.2016.00032 26904023 PMC4748049

[65] Hatfield SM Kjaergaard J Lukashev D Schreiber TH Belikoff B Abbott R. Immunological mechanisms of the antitumor effects of supplemental oxygenation. Sci Transl Med. (2015) 7:277ra30. 10.1126/scitranslmed.aaa1260 25739764 PMC4641038

[66] Jacobs SR Herman CE Maciver NJ Wofford JA Wieman HL Hammen JJ. Glucose uptake is limiting in T cell activation and requires CD28-mediated Akt-dependent and independent pathways. J Immunol (Baltimore, MD: 1950). (2008) 180:4476–86. 10.4049/jimmunol.180.7.4476 18354169 PMC2593791

[67] Wallace A Kapoor V Sun J Mrass P Weninger W Heitjan DF. Transforming growth factor-beta receptor blockade augments the effectiveness of adoptive T-cell therapy of established solid cancers. Clin Cancer Res. (2008) 14:3966–74. 10.1158/1078-0432.CCR-08-0356 18559619 PMC2491721

[68] Burga RA Thorn M Point GR Guha P Nguyen CT Licata LA. Liver myeloid-derived suppressor cells expand in response to liver metastases in mice and inhibit the anti-tumor efficacy of anti-CEA CAR-T. Cancer Immunol Immunother. (2015) 64:817–29. 10.1007/s00262-015-1692-6 25850344 PMC4485571

[69] Gabrilovich DI Nagaraj S. Myeloid-derived suppressor cells as regulators of the immune system. Nat Rev Immunol. (2009) 9:162–74. 10.1038/nri2506 19197294 PMC2828349

[70] Kang L Zhang J Li M Xu N Qi W Tan J. Characterization of novel dual tandem CD19/BCMA chimeric antigen receptor T cells to potentially treat multiple myeloma. Biomarker Res. (2020) 8:14. 10.1186/s40364-020-00192-6 32435496 PMC7222432

[71] Yan Z Cao J Cheng H Qiao J Zhang H Wang Y. A combination of humanised anti-CD19 and anti-BCMA CAR T cells in patients with relapsed or refractory multiple myeloma: a single-arm, phase 2 trial. Lancet Haematol. (2019) 6:e521–9. 10.1016/S2352-3026(19)30115-2 31378662

[72] Choi BD Yu X Castano AP Bouffard AA Schmidts A Larson RC. CAR-T cells secreting BiTEs circumvent antigen escape without detectable toxicity CAR-T cells secreting BiTEs circumvent antigen escape without detectable toxicity. Nat Biotechnol. (2019) 37:1049–58. 10.1038/s41587-019-0192-1 31332324

[73] Kumar V Patel S Tcyganov E Gabrilovich DI. The nature of myeloid-derived suppressor cells in the tumor microenvironment. Trends Immunol. (2016) 37:208–20. 10.1016/j.it.2016.01.004 26858199 PMC4775398

[74] Tesi RJ. MDSC; the most important cell you have never heard of. Trends Pharmacol Sci. (2019) 40:4–7. 10.1016/j.tips.2018.10.008 30527590

[75] Yang Z Guo J Weng L Tang W Jin S Ma W. Myeloid-derived suppressor cells-new and exciting players in lung cancer. J Hematol Oncol. (2020) 13:10. 10.1186/s13045-020-0843-1 32005273 PMC6995114

[76] Talmadge JE Gabrilovich DI. History of myeloid-derived suppressor cells. Nat Rev Cancer. (2013) 13:739–52. 10.1038/nrc3581 24060865 PMC4358792

[77] Pan PY Wang GX Yin B Ozao J Ku T Divino CM. Reversion of immune tolerance in advanced malignancy: modulation of myeloid-derived suppressor cell development by blockade of stem-cell factor function. Blood. (2008) 111:219–8. 10.1182/blood-2007-04-086835 17885078 PMC2200807

[78] Yang L Huang J Ren X Gorska AE Chytil A Aakre M. Abrogation of TGF beta signaling in mammary carcinomas recruits Gr-1+CD11b+ myeloid cells that promote metastasis. Cancer Cell. (2008) 13:23–35. 10.1016/j.ccr.2007.12.004 18167337 PMC2245859

[79] Sumida K Wakita D Narita Y Masuko K Terada S Watanabe K. Anti-IL-6 receptor mAb eliminates myeloid-derived suppressor cells and inhibits tumor growth by enhancing T-cell responses. Eur J Immunol. (2012) 42:2060–72. 10.1002/eji.201142335 22653638

[80] O'Neill LA Pearce EJ. Immunometabolism governs dendritic cell and macrophage function. J Exp Med. (2016) 213:15–23. 10.1084/jem.20151570 26694970 PMC4710204

[81] Liu M O'Connor RS Trefely S Graham K Snyder NW Beatty GL. Metabolic rewiring of macrophages by CpG potentiates clearance of cancer cells and overcomes tumor-expressed CD47-mediated ‘don't-eat-me' signal. Nat Immunol. (2019) 20:265–75. 10.1038/s41590-018-0292-y 30664738 PMC6380920

[82] Pollard JW Tumour-educated macrophages promote tumour progression and metastasis. Nat Rev Cancer. (2004) 4:71–8. 10.1038/nrc1256 14708027

[83] Cassetta L Pollard JW. Targeting macrophages: therapeutic approaches in cancer. Nat Rev Drug Discov. (2018) 17:887–904. 10.1038/nrd.2018.169 30361552

[84] Noy R Pollard JW. Tumor-associated macrophages: from mechanisms to therapy. Immunity. (2014) 41:49–61. 10.1016/j.immuni.2014.06.010 25035953 PMC4137410

[85] Tariq M Zhang J Liang G Ding L He Q Yang B. Macrophage polarization: anti-cancer strategies to target tumor-associated macrophage in breast cancer. J Cell Biochem. (2017) 118:2484–501. 10.1002/jcb.25895 28106295

[86] Taniguchi K Hikiji H Okinaga T Hashidate-Yoshida T Shindou H Ariyoshi W. Essential role of lysophosphatidylcholine acyltransferase 3 in the induction of macrophage polarization in PMA-treated U937 cells. J Cell Biochem. (2015) 116:2840–8. 10.1002/jcb.25230 25994902

[87] Tiainen S Tumelius R Rilla K Hamalainen K Tammi M Tammi R. High numbers of macrophages, especially M2-like (CD163-positive), correlate with hyaluronan accumulation and poor outcome in breast cancer. Histopathology. (2015) 66:873–3. 10.1111/his.12607 25387851

[88] Petty AJ Li A Wang X Dai R Heyman B Hsu D. Hedgehog signaling promotes tumor-associated macrophage polarization to suppress intratumoral CD8^+^ T cell recruitment. J Clin Investig. (2019) 129:5151–62. 10.1172/JCI128644 31638600 PMC6877305

[89] Huang YK Wang M Sun Y Di Costanzo N Mitchell C Achuthan A. Macrophage spatial heterogeneity in gastric cancer defined by multiplex immunohistochemistry. Nat Commun. (2019) 10:3928. 10.1038/s41467-019-11788-4 31477692 PMC6718690

[90] Goossens P Rodriguez-Vita J Etzerodt A Masse M Rastoin O Gouirand V. Membrane cholesterol efflux drives tumor-associated macrophage reprogramming and tumor progression. Cell Metab. (2019) 29:1376–89.e4. 10.1016/j.cmet.2019.02.016 30930171

[91] De Palma M Lewis CE. Macrophage regulation of tumor responses to anticancer therapies. Cancer Cell. (2013) 23:277–86. 10.1016/j.ccr.2013.02.013 23518347

[92] Cassier PA Italiano A Gomez-Roca CA Le Tourneau C Toulmonde M Cannarile MA. CSF1R inhibition with emactuzumab in locally advanced diffuse-type tenosynovial giant cell tumours of the soft tissue: a dose-escalation and dose-expansion phase 1 study. Lancet Oncol. (2015) 16:949–56. 10.1016/S1470-2045(15)00132-1 26179200

[93] Coniglio SJ Eugenin E Dobrenis K Stanley ER West BL Symons MH. Microglial stimulation of glioblastoma invasion involves epidermal growth factor receptor (EGFR) and colony stimulating factor 1 receptor (CSF-1R) signaling. Mol Med (Cambridge, Mass). (2012) 18:519–27. 10.2119/molmed.2011.00217 22294205 PMC3356419

[94] Yan D Kowal J Akkari L Schuhmacher AJ Huse JT West BL. Inhibition of colony stimulating factor-1 receptor abrogates microenvironment-mediated therapeutic resistance in gliomas. Oncogene. (2017) 36:6049–58. 10.1038/onc.2017.261 28759044 PMC5666319

[95] Cannarile MA Weisser M Jacob W Jegg AM Ries CH Rüttinger D. Colony-stimulating factor 1 receptor (CSF1R) inhibitors in cancer therapy. J Immunother Cancer. (2017) 5:53. 10.1186/s40425-017-0257-y 28716061 PMC5514481

[96] Butowski N Colman H De Groot JF Omuro AM Nayak L Wen PY. Orally administered colony stimulating factor 1 receptor inhibitor PLX3397 in recurrent glioblastoma: an Ivy Foundation Early Phase Clinical Trials Consortium phase II study. Neuro-oncology. (2016) 18:557–64. 10.1093/neuonc/nov245 26449250 PMC4799682

[97] Ries CH Cannarile MA Hoves S Benz J Wartha K Runza V. Targeting tumor-associated macrophages with anti-CSF-1R antibody reveals a strategy for cancer therapy. Cancer Cell. (2014) 25:846–59. 10.1016/j.ccr.2014.05.016 24898549

[98] Qian B Deng Y Im JH Muschel RJ Zou Y Li J. A distinct macrophage population mediates metastatic breast cancer cell extravasation, establishment and growth. PLoS ONE. (2009) 4:e6562. 10.1371/journal.pone.0006562 19668347 PMC2721818

[99] Hiraoka K Zenmyo M Watari K Iguchi H Fotovati A Kimura YN. Inhibition of bone and muscle metastases of lung cancer cells by a decrease in the number of monocytes/macrophages. Cancer Sci. (2008) 99:1595–02. 10.1111/j.1349-7006.2008.00880.x 18754872 PMC11158597

[100] Gazzaniga S Bravo AI A. Guglielmotti van Rooijen N Maschi F Vecchi A. Targeting tumor-associated macrophages and inhibition of MCP-1 reduce angiogenesis and tumor growth in a human melanoma xenograft. J Investig Dermatol. (2007) 127:2031–41. 10.1038/sj.jid.5700827 17460736

[101] Zhu Y Knolhoff BL Meyer MA Nywening TM West BL Luo J. CSF1/CSF1R blockade reprograms tumor-infiltrating macrophages and improves response to T-cell checkpoint immunotherapy in pancreatic cancer models. Cancer Res. (2014) 74:5057–69. 10.1158/0008-5472.CAN-13-3723 25082815 PMC4182950

[102] Li X Yao W Yuan Y Chen P Li B Li J. Targeting of tumour-infiltrating macrophages *via* CCL2/CCR2 signalling as a therapeutic strategy against hepatocellular carcinoma. Gut. (2017) 66:157–67. 10.1136/gutjnl-2015-310514 26452628

[103] Loberg RD Ying C Craig M Day LL Sargent E Neeley C. Targeting CCL2 with systemic delivery of neutralizing antibodies induces prostate cancer tumor regression *in vivo*. Cancer Res. (2007) 67:9417–24. 10.1158/0008-5472.CAN-07-1286 17909051

[104] Nywening TM Wang-Gillam A Sanford DE Belt BA Panni RZ Cusworth BM. Targeting tumour-associated macrophages with CCR2 inhibition in combination with FOLFIRINOX in patients with borderline resectable and locally advanced pancreatic cancer: a single-centre, open-label, dose-finding, non-randomised, phase 1b trial. Lancet Oncol. (2016) 17:651–2. 10.1016/S1470-2045(16)00078-4 27055731 PMC5407285

[105] Bonapace L Coissieux MM Wyckoff J Mertz KD Varga Z Junt T. Cessation of CCL2 inhibition accelerates breast cancer metastasis by promoting angiogenesis. Nature. (2014) 515:130–3. 10.1038/nature13862 25337873

[106] Arwert EN Harney AS Entenberg D Wang Y Sahai E Pollard JW. A unidirectional transition from migratory to perivascular macrophage is required for tumor cell intravasation. Cell Rep. (2018) 23:1239–248. 10.1016/j.celrep.2018.04.007 29719241 PMC5946803

[107] Marra M Salzano G Leonetti C Tassone P Scarsella M Zappavigna S. Nanotechnologies to use bisphosphonates as potent anticancer agents: the effects of zoledronic acid encapsulated into liposomes. Nanomed Nanotechnol Biol Med. (2011) 7:955–64. 10.1016/j.nano.2011.03.004 21453789

[108] Weiskopf K Ring AM Ho CC Volkmer JP Levin AM Volkmer AK. Engineered SIRPalpha variants as immunotherapeutic adjuvants to anticancer antibodies. Science (New York, NY). (2013) 341:88–91. 10.1126/science.1238856 23722425 PMC3810306

[109] Ruffell B Chang-Strachan D Chan V Rosenbusch A Ho CM Pryer N. Macrophage IL-10 blocks CD8^+^ T cell-dependent responses to chemotherapy by suppressing IL-12 expression in intratumoral dendritic cells. Cancer Cell. (2014) 26:623–37. 10.1016/j.ccell.2014.09.006 25446896 PMC4254570

[110] El Kasmi KC Qualls JE Pesce JT Smith AM Thompson RW Henao-Tamayo M. Toll-like receptor-induced arginase 1 in macrophages thwarts effective immunity against intracellular pathogens. Nat Immunol. (2008) 9:1399–406. 10.1038/ni.1671 18978793 PMC2584974

[111] Singh M Khong H Dai Z Huang XF Wargo JA Cooper ZA. Effective innate and adaptive antimelanoma immunity through localized TLR7/8 activation. J Immunol (Baltimore, MD: 1950). (2014) 193:4722–31. 10.4049/jimmunol.1401160 25252955 PMC4201984

[112] Le Mercier Poujol D Sanlaville A Sisirak V Gobert M Durand I. Tumor promotion by intratumoral plasmacytoid dendritic cells is reversed by TLR7 ligand treatment. Cancer Res. (2013) 73:4629–40. 10.1158/0008-5472.CAN-12-3058 23722543

[113] Smith DA Conkling P Richards DA Nemunaitis JJ Boyd TE Mita AC. Antitumor activity and safety of combination therapy with the Toll-like receptor 9 agonist IMO-2055, erlotinib, and bevacizumab in advanced or metastatic non-small cell lung cancer patients who have progressed following chemotherapy. Cancer Immunol Immunother. (2014) 63:787–96. 10.1007/s00262-014-1547-6 24770667 PMC11028443

[114] Vonderheide RH. CD40 Agonist antibodies in cancer immunotherapy. Ann Rev Med. (2019) 71:47–58. 10.1146/annurev-med-062518-045435 31412220

[115] Baer C Squadrito ML Laoui D Thompson D Hansen SK Kiialainen A. De palma, suppression of microRNA activity amplifies IFN-gamma-induced macrophage activation and promotes anti-tumour immunity. Nat Cell Biol. (2016) 18:790–802. 10.1038/ncb3371 27295554

[116] Guerriero JL Sotayo A Ponichtera HE Castrillon JA Pourzia AL Schad S. Class IIa HDAC inhibition reduces breast tumours and metastases through anti-tumour macrophages. Nature. (2017) 543:428–32. 10.1038/nature21409 28273064 PMC8170529

[117] Ding J Karp JE Emadi A. Elevated lactate dehydrogenase (LDH) can be a marker of immune suppression in cancer: interplay between hematologic and solid neoplastic clones and their microenvironments. Can Biomark A Dis Markers. (2017) 19:353–63. 10.3233/CBM-160336 28582845 PMC13020749

[118] Kaneda MM Messer KS Ralainirina N Li H Leem CJ Gorjestani S. PI3Kgamma is a molecular switch that controls immune suppression. Nature. (2016) 539:437–42. 10.1038/nature19834 27642729 PMC5479689

[119] Colegio OR Chu NQ Szabo AL Chu T Rhebergen AM Jairam V . Functional polarization of tumour-associated macrophages by tumour-derived lactic acid. Nature. (2014) 513:559–63. 10.1038/nature13490 25043024 PMC4301845

[120] Klichinsky M Ruella M Shestova O Lu XM Best A Zeeman M. Human chimeric antigen receptor macrophages for cancer immunotherapy. Nat Biotechnol. (2020) 38:947–953. 10.1038/s41587-020-0462-y 32361713 PMC7883632

[121] Zhang L Tian L Dai X Yu H Wang J Lei A. Pluripotent stem cell-derived CAR-macrophage cells with antigen-dependent anti-cancer cell functions. J Hematol Oncol. (2020) 13:153. 10.1186/s13045-020-00983-2 33176869 PMC7656711

[122] Ohue Y Nishikawa H. Regulatory T (Treg) cells in cancer: can Treg cells be a new therapeutic target? Cancer Sci. (2019) 110:2080–9. 10.1111/cas.14069 31102428 PMC6609813

[123] Khattri R Cox T Yasayko SA Ramsdell F. An essential role for scurfin in CD4^+^CD25^+^ T regulatory cells. Nat Immunol. (2003) 4:337–42. 10.1038/ni909 12612581

[124] Liu C Workman CJ Vignali DA. Targeting regulatory T cells in tumors. FEBS J. (2016) 283:2731–48. 10.1111/febs.13656 26787424

[125] Lu L Barbi J Pan F. The regulation of immune tolerance by FOXP3. Nat Rev Immunol. (2017) 17:703–17. 10.1038/nri.2017.75 28757603 PMC5793224

[126] Wing JB Tanaka A Sakaguchi S. Human FOXP3(+) regulatory T cell heterogeneity and function in autoimmunity and cancer. Immunity. (2019) 50:302–16. 10.1016/j.immuni.2019.01.020 30784578

[127] von Boehmer H Daniel C. Therapeutic opportunities for manipulating T(Reg) cells in autoimmunity and cancer. Nat Rev Drug Discov. (2013) 12:51–63. 10.1038/nrd3683 23274471

[128] Zhang R Huynh A Whitcher G Chang J Maltzman JS Turka LA. An obligate cell-intrinsic function for CD28 in Tregs. J Clin Investig. (2013) 123:580–93. 10.1172/JCI65013 23281398 PMC3561819

[129] Foss F. Clinical experience with denileukin diftitox (ONTAK). Sem Oncol. (2006) 33:S11–6. 10.1053/j.seminoncol.2005.12.017 16516670

[130] Rech AJ Mick R Martin S Recio A Aqui NA Powell DJ. CD25 blockade depletes and selectively reprograms regulatory T cells in concert with immunotherapy in cancer patients. Sci Transl Med. (2012) 4:134ra62. 10.1126/scitranslmed.3003330 22593175 PMC4425934

[131] Sakaguchi S Sakaguchi N Asano M Itoh M Toda M. Immunologic self-tolerance maintained by activated T cells expressing IL-2 receptor alpha-chains (CD25). Breakdown of a single mechanism of self-tolerance causes various autoimmune diseases. J Immunol (Baltimore, MD: 1950). (1995) 155:1151–64. 7636184

[132] Langhans B Nischalke HD Kramer B Dold L Lutz P Mohr R. Role of regulatory T cells and checkpoint inhibition in hepatocellular carcinoma. Cancer Immunol Immunother. (2019) 65:2055–66. 10.1007/s00262-019-02427-4 31724091 PMC11028391

[133] Schaer DA Budhu S Liu C Bryson C Malandro N Cohen A. GITR pathway activation abrogates tumor immune suppression through loss of regulatory T cell lineage stability. Cancer Immunol Res. (2013) 1:320–1. 10.1158/2326-6066.CIR-13-0086 24416730 PMC3885345

[134] Tkachev V Furlan SN Watkins B Hunt DJ Zheng HB Panoskaltsis-Mortari A. Combined OX40L and mTOR blockade controls effector T cell activation while preserving Treg reconstitution after transplant. Sci Transl Med. (2017) 9:aan3085. 10.1126/scitranslmed.aan3085 28931653 PMC5681253

[135] Berlato C Khan MN Schioppa T Thompson R Maniati E Montfort A. A CCR4 antagonist reverses the tumor-promoting microenvironment of renal cancer. J Clin Investig. (2017) 127:801–13. 10.1172/JCI82976 28134623 PMC5330727

[136] Barsheshet Y Wildbaum G Levy E Vitenshtein A Akinseye C Griggs J. CCR8(+)FOXp3(+) Treg cells as master drivers of immune regulation. Proc Natl Acad Sci USA. (2017) 114:6086–91. 10.1073/pnas.1621280114 28533380 PMC5468670

[137] Eksteen B Miles A Curbishley SM Tselepis C Grant AJ Walker LS. Epithelial inflammation is associated with CCL28 production and the recruitment of regulatory T cells expressing CCR10. J Immunol (Baltimore, MD: 1950). (2006) 177:593–603. 10.4049/jimmunol.177.1.593 16785557

[138] Hansen W Hutzler M Abel S Alter C Stockmann C Kliche S. Neuropilin 1 deficiency on CD4^+^Foxp3^+^ regulatory T cells impairs mouse melanoma growth. J Exp Med. (2012) 209:2001–16. 10.1084/jem.20111497 23045606 PMC3478934

[139] Chapman NM Chi H. mTOR signaling, Tregs and immune modulation. Immunotherapy. (2014) 6:1295–311. 10.2217/imt.14.84 25524385 PMC4291176

[140] Newton R Priyadharshini B Turka LA. Immunometabolism of regulatory T cells. Nat Immunol. (2016) 17:618–25. 10.1038/ni.3466 27196520 PMC5006394

[141] Gerriets VA Kishton RJ Johnson MO Cohen S Siska PJ Nichols AG. Foxp3 and Toll-like receptor signaling balance Treg cell anabolic metabolism for suppression. Nat Immunol. (2016) 17:1459–66. 10.1038/ni.3577 27695003 PMC5215903

[142] Chen X Wei M Liu X Song S Wang L Yang X. Construction and validation of the CRISPR/dCas9-EZH2 system for targeted H3K27Me3 modification. Biochem Biophys Res Commun. (2019) 511:246–52. 10.1016/j.bbrc.2019.02.011 30795863

[143] Moreno Ayala MA Li Z DuPage M. Treg programming and therapeutic reprogramming in cancer. Immunology. (2019) 157:198–209. 10.1111/imm.13058 30866047 PMC6587317

[144] Allan SE Song-Zhao GX Abraham T McMurchy AN Levings MK. Inducible reprogramming of human T cells into Treg cells by a conditionally active form of FOXP3. Eur J Immunol. (2008) 38:3282–9. 10.1002/eji.200838373 19039775

[145] Yang R Qu C Zhou Y Konkel JE Shi S Liu Y. Hydrogen sulfide promotes Tet1- and Tet2-mediated Foxp3 demethylation to drive regulatory T cell differentiation and maintain immune homeostasis. Immunity. (2015) 43:251–63. 10.1016/j.immuni.2015.07.017 26275994 PMC4731232

[146] Ghosh S Taylor A Chin M Huang HR Conery AR Mertz JA. Regulatory T cell modulation by CBP/EP300 bromodomain inhibition. J Biol Chem. (2016) 291:13014–27. 10.1074/jbc.M115.708560 27056325 PMC4933219

[147] Di Pilato M Kim EY Cadilha BL Prussmann JN Nasrallah MN Seruggia D. Targeting the CBM complex causes Treg cells to prime tumours for immune checkpoint therapy. Nature. (2019) 570:112–6. 10.1038/s41586-019-1215-2 31092922 PMC6656391

[148] Kim HJ Barnitz RA Kreslavsky T Brown FD Moffett H Lemieux ME. Stable inhibitory activity of regulatory T cells requires the transcription factor Helios. Science (New York, NY). (2015) 350:334–9. 10.1126/science.aad0616 26472910 PMC4627635

[149] Sharma MD Huang L Choi JH Lee EJ Wilson JM Lemos H. An inherently bifunctional subset of Foxp3^+^ T helper cells is controlled by the transcription factor eos. Immunity. (2013) 38:998–1012. 10.1016/j.immuni.2013.01.013 23684987 PMC3681093

[150] Kerdiles YM Stone EL Beisner DR McGargill MA Ch'en IL Stockmann C. Foxo transcription factors control regulatory T cell development and function. Immunity. (2010) 33:890–904. 10.1016/j.immuni.2010.12.002 21167754 PMC3034255

[151] Wang D Quiros J Mahuron K Pai CC Ranzani V Young A. Targeting EZH2 reprograms intratumoral regulatory T cells to enhance cancer immunity. Cell Rep. (2018) 23:3262–74. 10.1016/j.celrep.2018.05.050 29898397 PMC6094952

[152] Fang F Xiao W Tian Z. NK cell-based immunotherapy for cancer. Sem Immunol. (2017) 31:37–54. 10.1016/j.smim.2017.07.009 28838796

[153] Muntasell A Ochoa MC Cordeiro L Berraondo P Lopez-Diaz de Cerio A. Targeting NK-cell checkpoints for cancer immunotherapy. Curr Opin Immunol. (2017) 45:73–81. 10.1016/j.coi.2017.01.003 28236750

[154] Vivier E Ugolini S Blaise D Chabannon C Brossay L. Targeting natural killer cells and natural killer T cells in cancer. Nat Rev. Immunol. (2012) 12:239–52. 10.1038/nri3174 22437937 PMC5161343

[155] Barrow AD Edeling MA Trifonov V Luo J Goyal P Bohl B. Natural killer cells control tumor growth by sensing a growth factor. Cell. (2018) 172:534–48.e19. 10.1016/j.cell.2017.11.037 29275861 PMC6684025

[156] Rezvani K Rouce R Liu E Shpall E. Engineering natural killer cells for cancer immunotherapy. Mol Ther J Am Soc Gene Ther. (2017) 25:1769–81. 10.1016/j.ymthe.2017.06.012 28668320 PMC5542803

[157] Pahl J Cerwenka A. Tricking the balance: NK cells in anti-cancer immunity. Immunobiology. (2017) 222:11–20. 10.1016/j.imbio.2015.07.012 26264743

[158] Floros T Tarhini AA. Anticancer cytokines: biology and clinical effects of interferon-alpha2, interleukin (IL)-2, IL-15, IL-21, and IL-12. Sem Oncol. (2015) 42:539–48. 10.1053/j.seminoncol.2015.05.015 26320059 PMC4557618

[159] Pillet AH Theze J Rose T. Interleukin (IL)-2 and IL-15 have different effects on human natural killer lymphocytes. Hum Immunol. (2011) 72:1013–7. 10.1016/j.humimm.2011.07.311 21925225

[160] Srivastava S Pelloso D Feng H Voiles L Lewis D Haskova Z. Effects of interleukin-18 on natural killer cells: costimulation of activation through Fc receptors for immunoglobulin. Cancer Immunol Immunother. (2013) 62:1073–82. 10.1007/s00262-013-1403-0 23604103 PMC3707624

[161] Becker PS Suck G Nowakowska P Ullrich E Seifried E Bader P. Selection and expansion of natural killer cells for NK cell-based immunotherapy. Cancer Immunol Immunother. (2016) 65:477–84. 10.1007/s00262-016-1792-y 26810567 PMC4826432

[162] Hu Y Tian ZG Zhang C. Chimeric antigen receptor (CAR)-transduced natural killer cells in tumor immunotherapy. Acta Pharmacologica Sinica. (2018) 39:167–76. 10.1038/aps.2017.125 28880014 PMC5800464

[163] Shaim H Yvon E. Cord blood: a promising source of allogeneic natural killer cells for immunotherapy. Cytotherapy. (2015) 17:1–2. 10.1016/j.jcyt.2014.12.001 25527863

[164] Verneris MR Miller JS. The phenotypic and functional characteristics of umbilical cord blood and peripheral blood natural killer cells. Br J Haematol. (2009) 147:185–91. 10.1111/j.1365-2141.2009.07768.x 19796267 PMC2770803

[165] Oelsner S Friede ME Zhang C Wagner J Badura S Bader P. Continuously expanding CAR NK-92 cells display selective cytotoxicity against B-cell leukemia and lymphoma. Cytotherapy. (2017) 19:235–49. 10.1016/j.jcyt.2016.10.009 27887866

[166] Romanski A Uherek C Bug G Seifried E Klingemann H Wels WS. CD19-CAR engineered NK-92 cells are sufficient to overcome NK cell resistance in B-cell malignancies. J Cell Mol Med. (2016) 20:1287–94. 10.1111/jcmm.12810 27008316 PMC4929308

[167] Zhang C Burger MC Jennewein L Genssler S Schonfeld K Zeiner P. ErbB2/HER2-Specific NK cells for targeted therapy of glioblastoma. J Natl Cancer Inst. (2016) 108. 10.1093/jnci/djv375 26640245

